# Repeated resection-associated breast angiosarcoma: A case report

**DOI:** 10.1097/MD.0000000000012513

**Published:** 2018-09-28

**Authors:** Tiantian Tang, Haiping Li

**Affiliations:** Breast Center, The Fourth Hospital of Hebei Medical University, Shijiazhuang City, Hebei Province, P. R. China.

**Keywords:** angiosarcoma, breast, cause, repeated resection, therapy

## Abstract

**Rationale:**

As a very rare vascular tumor, breast angiosarcoma (AS) can be divided into primary and second breast AS. However, the latter is slightly more commonly detected in clinical practice. Radiation post mastectomy is the common cause for the secondary breast AS, and although there are other reasons, it is still quite rare. In the present study, we reported a rare case of breast AS and summarized the relevant literatures so that to conduce to diagnose AS.

**Patient concerns:**

A 50-year-old female with a history of right breast neoplasm was treated with repeat lumpectomy for 4 times during 8 years.

**Diagnoses:**

Mammogram and ultrasound examination demonstrated a possible malignancy (BIRADS-4B and BI-RADS-4C, respectively). Immunohistochemically positive for endothelial markers CD31, CD34, ERG, and FVIII-R-Ag.

**Interventions:**

The patient underwent a right mastectomy with sentinel lymph node biopsy by our multidisciplinary team and no other therapy was given postsurgery.

**Outcomes:**

The patient had no recurrence after 3 months.

**Lessons:**

Based on our findings, we concluded that repeated resection might be a risk factor for the breast AS, especially for a gradual pathological evolution from benign to malignant. This case showed a very rare cause for angiomatosis of breast, and the patient had a successful outcome after a simple mastectomy.

## 1 Introduction

As a rare vascular tumor, angiosarcoma (AS) originates from vascular channels lined by endothelial cells (endoderm tumor). Its incidence is around 0.0005% to 0.05% in all malignant breast neoplasms.^[1–3]^ AS can be divided into primary and secondary subtypes, and most of secondary AS are known to be induced by radiation. Normally, secondary AS is found in older women who have undergone breast conserving surgery (BCS) and radiation therapy (RT) or chest wall RT.^[4–6]^ In the present study, we reported a rare case of breast AS and summarized the relevant literatures.

## 2 Case report

A 50-year-old Chinese female with a history of right breast neoplasm was treated with repeat lumpectomy for 4 times during 8 years. Physical examination revealed a 2.0-cm palpable mass in the right breast at the original surgical site. The lesion was not associated with any edema or blister, and no skin discoloration or ulcer was found. Subsequent mammogram and ultrasound examination demonstrated a possible malignancy (BI-RADS-4B and BI-RADS-4C, respectively). Digital mammography and ultrasonography indicated that there were no other specific characteristics compared with breast cancer. A CT scan for brain, lung, liver, and bone were conducted to exclude metastasis.

**Figure 1:**
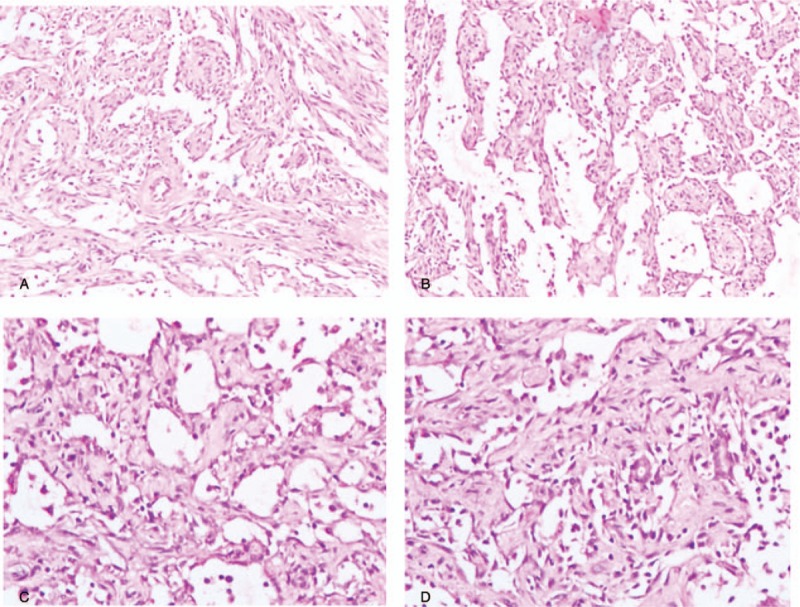
A and B, Original magnification ×100: HE staining showed tumor cells with a solid distribution, forming by irregular capillary lumen, which was anastomosed each other. C and D, Original magnification ×200: HE staining showed that endothelial cells were in spindle or polygon shape. Diffuse hyperplasia, irregular shape, and a small amount of red blood cells could be seen in the vascular cavity.

**Figure 2:**
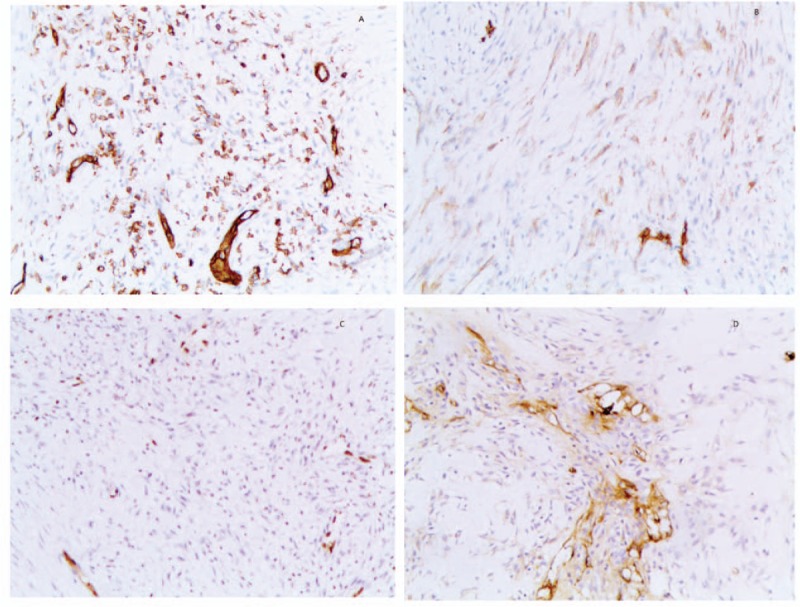
A, Special staining of CD31 by IHC analysis. B, A special staining of CD34 by IHC analysis. C, A special staining of ERG by IHC analysis. D, A special staining of FVIII-R-Ag by IHC analysis. ERG = ETS related gene, IHC = immunohistochemical.

The patient had a history of repeat lumpectomy, and the pathological analysis showed adenosis, phyllodes tumor, and fibrous tissue, accompanied by glass changes and highly differentiated AS. Therefore, mastectomy was suggested by our multidisciplinary team, and the patient underwent a right mastectomy with sentinel lymph node biopsy in January, 2018. The lesion was diagnosed as AS (Fig. 1A–D), which was immunohistochemically positive for endothelial markers CD31 (Fig. 2A), CD34 (Fig. 2B), ETS related gene (Fig. 2C), and FVIII-R-Ag (Fig. 2D). No other therapy was given postsurgery, and the patient had no recurrence after 3 months. This study was proved by the Ethical Committee of our hospital, and a written consent was obtained from the patient.

## 3 Discussion

Primary breast AS accounts for <0.1% of all malignant breast tumors, and no recognizable risk factor is available for its diagnosis. It typically occurs within breast parenchyma in women aged between 20 and 40 years.^[7]^ The morbidity of secondary breast AS is about <0.5%, and it is usually detected in elderly females between 56 and 89 years with presumed etiological factors.^[8]^ Common risk factors include BCS, RT, mastectomy, and axillary dissection.^[9]^ As for this patient, 4 times of lumpectomy might largely contribute to her AS, especially for a gradual pathological evolution from benign to malignant in this patient.

Most breast AS presents a painless cutaneous lesion and reddish purplish coloration.^[10]^ It may be multifocal, typically involving a significant part of the breast, and it is often associated with swelling, skin dimpling, and thickening. The tumor size may vary and has been reported to range from 0.4 to 20 cm, with a mean tumor size of 7.5 cm.^[7]^

In this case report, the clinical presentation was somewhat atypical as the tumor was small. It was not associated with edema or purple skin discoloration, and only low proliferation was detected.

It is difficult to distinct breast AS and breast cancer by mammography and ultrasound examination. However, magnetic resonance imaging is more likely to reveal the typical malignant signs for breast AS with low diagnostic sensitivity.^[10]^ Core biopsy is a useful approach in diagnosis of breast AS, especially when there is excessive bleeding during core needle biopsy. However, the accurate diagnosis depends on excisional biopsy. Through immunohistochemical (IHC) analysis, CD31, factor VIII, CD34, and Fli1 positivity can be used to confirm the diagnosis.^[11]^

Lymphatic spread of AS is less commonly observed.^[12]^ It often spreads through hematogenous route, and its most common sites of metastasis are lungs. Surgery is the main therapeutic scheme for all resectable tumors. Total mastectomy with or without axillary dissection is the preferred surgical method because of less lymphatic expansion. Routine use of axillary lymph node dissection is debatable, as most studies have shown that axillary lymph node positivity is less than 10%.^[13]^ The role of chemotherapy and radiotherapy in the adjuvant setting is also controversial. A retrospective observational study shows that the disease-free interval with adjuvant treatment is 9 to 12 months.^[14]^ It is not suggested to receive radiotherapy in the adjuvant setting since the evidence is limited. However, all currently available data are obtained based on small retrospective studies. Some patients can achieve disease control though adjuvant radiotherapy after mastectomy, while other studies have found that some patients still have recurrence in a form of lung or liver metastasis even when patients have received adjuvant radiotherapy.^[14]^

Most studies have shown the poor prognosis for AS, though cases are rare. In addition, the 5-year overall survival for primary breast AS and secondary AS is 44% and 69%, respectively.^[15,16]^

There are some limitations in our current case report. The major one is that there was no pathological confirmation before the surgery. Theoretically, a pathological confirmation should be conducted before any mastectomy.

## 4 Conclusions

Breast AS is a rare tumor. RT postmastectomy is thought to be the common reason for secondary breast AS. This report showed that a rare factor might probably cause breast AS. Considering her history of 4 times of surgery, we concluded that repeated resection might be a risk factor for breast AS, especially for a pathological evolution from benign to malignant.

## Author contributions

**Project administration:** Haiping Li.

**Writing – original draft:** Tiantian Tang.

**Writing – review & editing:** Tiantian Tang.
